# Second Primary Malignancies in Patients with Differentiated Thyroid Cancer after Radionuclide Therapy: A Retrospective Single-Centre Study

**DOI:** 10.3390/curroncol30010003

**Published:** 2022-12-20

**Authors:** Leandra Piscopo, Fabio Volpe, Carmela Nappi, Emilia Zampella, Mariarosaria Manganelli, Francesca Matrisciano, Pasquale Totaro, Leonardo Pace, Simone Maurea, Alberto Cuocolo, Michele Klain

**Affiliations:** 1 Department of Advanced Biomedical Sciences, University Federico II, 80131 Naples, Italy; 2 Department of Medicine, Surgery and Dentistry, University of Salerno, 84084 Fisciano, Italy

**Keywords:** differentiated thyroid cancer, second primary malignancies, radioactive iodine

## Abstract

Second primary malignancies (SPM) are described as any primary, not synchronous, malignancy arising in a different anatomical district, with confirmed histological diagnosis. Age at diagnosis, previous non-thyroidal primary malignancy, and radioactive iodine (RAI) therapy have been proposed as independent risk factors for SPM. RAI therapy is a standard treatment for moderate-high risk differentiated thyroid cancer (DTC), and its effect on the development of SPM has become a critical topic in DTC treatment. The purpose of this retrospective single-center study was to investigate the occurrence and the possible association of non-thyroidal SPM diagnosed after DTC and RAI therapy in a cohort of 1326 consecutive DTC patients referred at our Institution for RAI treatment from 1993 to 2009. Eighty-nine patients with ages ≤ 18 years at the time of DTC diagnosis or with a follow-up of ≤12 months were excluded from the final analysis. All patients underwent a complete clinical and hematological follow-up every 6 months for a minimum of 12 months. During follow-up (mean 89 ± 73 months), 25 patients (2%) had an SPM diagnosis (mean 133 ± 73 months). The most common site of the second malignancy was the breast, accounting for 32% of all SPM, followed by colon-rectal cancer (16%), leukemia, and gynecological and kidney cancer (4%). At Cox univariable regression analysis, age at DTC diagnosis (*p* < 0.001), age ≥55 years (*p* < 0.001) and follow-up duration (*p* < 0.004) were associated with SPM onset, while no significant association was observed with the administered activity of radioiodine. In conclusion, our data suggest that the older a person gets, the more sharply the likelihood of developing additional diseases, such as PMS, increases. Similarly, for follow-up, the more a patient is followed up clinically over time, the higher the risk of new diagnoses increases.

## 1. Introduction

Differentiated thyroid cancer (DTC) is estimated to be the sixth most common cancer in 2021, with incidence rates of 10.1 per 100,000 women and 3.1 per 100,000 men and mortality rates of 0.5 per 100,000 women and 0.3 per 100,000 men [1,2,3,4,5,6]. The diagnosis of DTC is rapidly rising with small papillary thyroid carcinoma, with an improved prognosis [1,2,3,4,5,6]. The survival rate of DTC in Italy improved from 91% to 99% (from 1970 to 2004) [5].

Second primary malignancies (SPM), described as any primary, not synchronous, malignancy arising in a different anatomical district with confirmed histological diagnosis, is reported in patients with DTC [5]. Actually, different incidences of SPM have been previously reported, ranging from 0.5% to 22% [7,8,9,10,11,12,13,14,15,16,17,18,19,20]. Moreover, several factors can influence the incidence of SPM in patients with DTC. The time elapsed between DTC diagnosis and SPM has proved to be relevant, varying from 2 months up to over 2 years of follow-up, with the risk of SPM being higher in DTC survivors compared to the cancer risk in the general population [5]. Age at diagnosis, previous non-thyroidal primary malignancy, and radioactive iodine (RAI) therapy have been proposed as independent risk factors for SPM [5]. RAI therapy is a standard treatment for moderate-high-risk DTC [21,22,23], and its effect on the development of SPM has become a critical topic in DTC treatment. An increased risk of SPM was reported in a large-scale cohort study, but the development of SPM after RAI administration was not clearly demonstrable [24]. While some studies report that a higher SPM risk is associated particularly with high cumulative ^131^I doses [7,8,9,10], in other studies, no association between RAI therapy and the risk of SPM was found [13,25,26]. Hence, the relationship between RAI therapy and SPM remains controversial.

The purpose of this study was to investigate the occurrence and the possible association of non-thyroidal SPM diagnosed after DTC and RAI therapy in a retrospective cohort study.

## 2. Materials and Methods

### 2.1. Study Cohort

We performed a retrospective single-center study in a cohort of 1326 consecutive DTC patients who were referred to our Institution for RAI treatment and follow-up of DTC from 1993 to 2009. Demographic, clinical, surgical, and pathological data were noted from clinical records, including the age at the time of DTC diagnosis, the extent of surgery, the administered RAI dose, and the latest date of follow-up. According to the American Thyroid Association risk stratification [6], the activity administered in low-risk patients ranged from 1110 to 2220 MBq, in intermediate-risk patients from 2479 to 3700 MBq and in high-risk patients from 4440 to 7400 MBq.

### 2.2. Follow-Up

All patients underwent a complete clinical and hematological follow-up every 6 months for a minimum of 12 months (mean 89 ± 73 months). SPM was described as any primary, not synchronous, malignancy arising in a different anatomical district with a confirmed histological diagnosis. The timing of SPM occurrence with respect to DTC diagnosis was chosen to be more than 12 months after DTC. The SPM diagnosed within 12 months of RAI administration was considered synchronous and excluded from our analysis. The date of recurrence, SPM diagnosis and most recent outpatient visit were recorded as follow-up duration. If patient death occurred during follow-up, it was noted.

### 2.3. Statistical Analysis

Continuous data are expressed as mean ± standard deviation and categorical data as a percentage. Comparison between groups was performed with unpaired *t*-test and Chi-square test as appropriate. A *p*-value < 0.05 was considered statistically significant. Univariable Cox regression analysis was performed for each variable. Only variables showing a *p*-value < 0.05 at univariable analysis were considered statistically significant. Statistical analysis was performed with Stata 15.1 software (StataCorp, College Station, TX, USA).

## 3. Results

**Figure 1:**
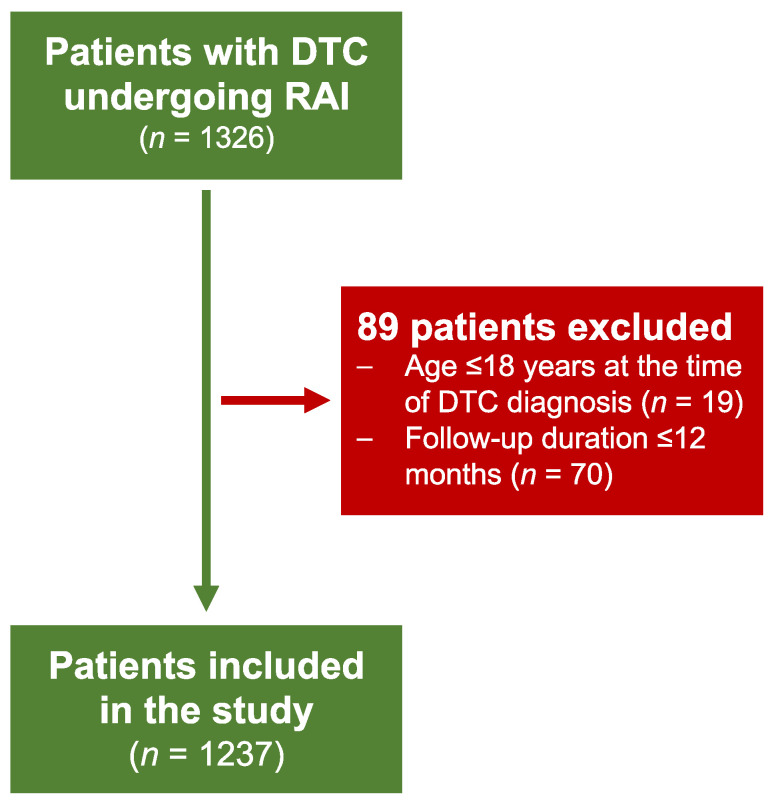
Studied population.

None of the patients potentially eligible for the study refused to participate, and all patients were able to sustain the examination. From the 1326 patients available from the analysis, 89 subjects with age ≤ 18 years at the time of DTC diagnosis or with a follow-up ≤12 months were excluded from the final analysis leaving 1237 patients (985 women, 252 men; mean age of 45 ± 14) for the final analysis (Figure 1).

Baseline characteristics of the patient population, histological characteristics, follow-up duration and dose administration, are summarized in Table 1.

The mean initial RAI dose is 3552 ± 1110 MBq The mean number of treatments is 1.44 ± 1.067, and the average treatment interval is 25 ± 2 months.

As shown in Table 1, during follow-up (mean 89 ± 73 months), 25 patients (2%) had an SPM diagnosis (mean 133 ± 73 months). The mean age of these patients (55 ± 16 years) was lower compared to those without SPM (45 ± 14, *p* < 0.0001), while no differences in gender and in histological type were found (Table 1). Moreover, no significant difference was observed in the total administered ^131^I activity (6364 ± 5176 MBq vs. 5710 ± 5820 MBq). Interestingly, follow-up duration was significantly longer in patients experiencing SPM compared to those without documented SPM (133 ± 73 MBq vs. 89 ± 73, *p* = 0.003).

SPM histology and number are described in Table 2, along with the ^131^I activity administered. The most common site of the SPM was the breast, accounting for 32% of all SPM, followed by colon-rectal cancer (16%), leukemia, and gynecological and kidney cancer (4%). To note, only one out of the eight female patients developing breast cancer was of childbearing age, while the other seven were in postmenopausal age at the time of RAI, further supporting data that excludes a potential relationship between RAI and breast cancer development.

At Cox univariable regression analysis, as described in Table 3, increasing age at the DTC diagnosis (*p* < 0.001) and follow-up duration (*p* < 0.005) were the only factors significantly associated with SPM onset.

Of note, only 11 out of 25 patients showed SPM. By mean, 44% of the subjects with the event had the secondary malignancy diagnosis within five years of RAI therapy.

At the end of the follow-up, 25 patients had died. Among these, 2 patients died due to SPM, and 23 patients died due to non-oncological reasons. There were 20 patients who died from cardiovascular events and 3 patients who died from infection accidents.

## 4. Discussion

In the present study, a relatively low percentage of patients (2%) showed SPM during follow-up. According to data available from the Italian Tumor Registry Association (AIRTUM) on https://www.registri-tumori.it/cms (accessed on 1 September 2022), it has been reported that the number of observed second cancers in patients with DTC in the Italian population is 404/12,866 patients overall, with a frequency of 3% of patients showing an SPM after DTC. Our findings are thus in line with Italian data. Age at DTC diagnosis and duration of follow-up were the only significant factors associated with the development of SPM after RAI. This finding is not surprising given the excellent long-life expectancy of the investigated population. It becomes clear that a longer follow-up certainly has a better chance of bringing out new diagnoses such as SPMs, especially in patients on surveillance programs, as in the case of patients with DTC [12,13,15].

Discordant data regarding the development of SPM in patients treated with RAI have been reported [7,8,9,10,11,12,13,14,15,16,17,18,19,20,27]. Undeniably, the body of evidence provided by literature on this topic shows a wide heterogeneity in methods and results [7,8,9,10,11,12,13,14,15,16,17,18,19,20,27]. To date, the risk of developing SPM after an RAI cannot be excluded a priori, but it is expected to be low [10,11,12,17,18,20].

Our findings are consistent with those from Cappagli et al. [13], who retrospectively evaluated the risk of developing SPM in 1096 consecutive DTC patients after RAI treatment, observing that only 7% of patients experienced an SPM. This investigation demonstrates that while the cumulative dose of ^131^I is not significantly associated with the development of an SPM, only the duration of follow-up shows a significant relationship with the risk of an SPM after DTC supporting the idea that a long follow-up determines more effective health surveillance including SPM diagnosis. Likewise, Mei et al. [12] found that 4% of 104,026 DTC survivors developed SPM during the follow-up. The overall risk of SPM associated with RAI increased with age at DTC diagnosis and decreased after two years from RAI [12].

Furthermore, a recent meta-analysis evaluated the risk of breast cancer in patients with DTC receiving RAI taking into consideration 14 studies [26] and reported that the pooled risk of primary breast cancer after a long-term follow-up is not higher than the risk observed in those not treated with RAI. [26].

Crocetti et al. [25], found that the standardized incidence ratio of SPM on an Italian cohort of 276,100 patients with DTC not treated with RAI was 1.16. This finding is consistent with the results of the present investigation carried out in a population who underwent RAI therapy, suggesting that this treatment does not influence the risk of SPM.

On the other hand, Brown et al. [15] evaluated a total of 30,278 DTC patients, for a median follow-up of 103 months, with 2158 (7%) showing SPM. A greater significant risk of SPM was found for patients treated with RAI as compared to those not treated. Similarly, Rubino et al. [8] evaluated a European cohort of 6841 DTC patients with a mean age of 44 years, with an overall increased risk of SPM of 27% as compared to the general population. An increased risk of SPM was associated with the increasing cumulative activity of ^131^I administered.

Thus, on the one hand, our data demonstrate a low number of SPM. On the other, larger cohort studies have reported higher rates of SPM. However, given the observational retrospective nature of the present analysis, some events would have been missed on follow-up, explaining a slight underestimation of SMPs in our investigation. Indeed, within DTC follow-up, the enrolled patients were not routinely referred to clinical and imaging tests that were not directly related to DTC surveillance.

Hong et al. [27], in a multicenter retrospective study, analyzed a total of 3106 DTC patients who underwent RAI. A total of 183 SPM, which included 162 solid and 21 hematologic cancers, occurred in 173 patients (5.6%). A multivariate analysis identified independent prognostic factors for the development of SPM, including age at RAI, male gender, and total cumulative dose over 200 mCi.

The incidence of SPM in the present study is very low compared to that found in some studies described in the literature [7,8,13,14,15,19]. This difference could be explained by considering the characteristics of the current study population, such as the exclusion of pediatric patients, with a more aggressive tumor phenotype and the follow-up length.

For some authors [12,13,15], the latency time is an important factor that influences SPM development. However, in our sample, only 44% of the subjects with SPM had the secondary malignancy diagnosis within five years of the RAI therapy.

Another factor worth considering is the ^131^I cumulative activity dose. The present investigation demonstrates that ^131^I cumulative activity dose is not associated with SPM development in DTC patients after RAI therapy, even considering different administered activity categories. This finding is in agreement with numerous prior studies [5,11,12,13,14,19,20,27,28,29,30]. However, in the study by Hong et al. [27] total cumulative dose over 200 mCi was significantly associated with SPM development. In the same way, Lang et al. [7] and Rubino et al. [8] found an increased risk of SPM with the growing cumulative activity of ^131^I administered. Therefore, the association between RAI and the risk of SPM development in DTC patients is still controversial. It is certainly very important that every patient must be contextualized and deserves a personal medical evaluation based on their genetic and clinical background [5,7,8,9,10,11,12,13,14,15,16,17,18,19,20]. With regard to women, in the studied population, only one out of the eight female patients developing breast cancer was of childbearing age, while the other seven were in postmenopausal age at the time of RAI, further supporting data that excludes a potential relationship between RAI and breast cancer development. This finding is consistent with a recent meta-analysis [26] demonstrating that patients with DTC treated with RAI do not have a higher risk of primary breast cancer compared to those not treated with RAI.

The results of our study suggest that the older a person gets, the more sharply the likelihood of developing additional diseases, such as SPM, increases.

In this context, it should be considered that the pediatric population has been excluded from the final analysis in light of known differences between adult and pediatric DTC regarding clinical, molecular, and pathological characteristics [31]. Thus, specific investigations for the pediatric population are needed. We excluded such a population that will be the object of a different study to avoid any bias in the results interpretation.

Similarly, for follow-up, the more a patient is followed up clinically over time, the higher the risk of new diagnoses increases.

## 5. Conclusions

In the present study, only increasing age at the DTC diagnosis and follow-up length were significantly associated with SPM onset. On the contrary, ^131^I cumulative activity was not associated with SPM development. Breast cancer was the most common secondary non-thyroid cancer. Our data can also be read in light of the long-life expectancy of patients with DTC, making the malignancy rate of this patient category like general population findings. The high incidence of breast cancer in our cohort of patients can be explained by the high prevalence (i.e., 80%) of women enrolled.

## Figures and Tables

**Table 1:** Clinical characteristics in the overall patient population according to SPM occurrence.

	All Patients (*n* = 1237)	Without SPM (*n* = 1212)	With SPM (*n* = 25)	*p* Value
Age (years)	45 ± 14	45 ± 14	55 ± 16	0.001
Age ≥ 55 years	338 (27)	323 (27)	15 (60)	0.001
Female gender, *n* (%)	985 (80)	967 (80)	18 (72)	0.34
Papillary type, *n* (%)	641 (52)	627 (52)	14 (56)	0.67
>T2, *n* (%)	206 (87)	200 (16)	6 (24)	0.17
N1, *n* (%)	203 (16)	203 (17)	0 (0)	0.02
^131^I cumulative activity (MBq)	5722 ± 5867	5710 ± 5820	6364 ± 5176	0.58
≤1850 MBq, *n* (%)	131 (11)	130 (11)	1 (4)	0.28
1850–3700 MBq, *n* (%)	693 (56)	682 (56)	11 (44)	0.22
>3700 MBq, *n* (%)	413 (33)	400 (33)	13 (52)	0.05
Follow-up duration (months)	89 ± 73	89 ± 73	133 ± 73	0.001

Data are presented as the mean value ± SD or number and percentage (%).

**Table 2:** Observed second primary malignancies and RAI activity in 25 patients.

Cancer Type	SPM, *n* (%)	Amount of ^131^I (MBq)
Breast	8 (32)	6532
Colon and rectum	4 (16)	3700
Lung	1 (4)	24,050
Leukemia	2 (8)	5143
Kidney	2 (8)	5162
Cholangiocarcinoma	1 (4)	4366
Meningioma	1 (4)	11,100
Salivary glands	1 (4)	4366
Gynecological	2 (8)	2997
Prostate	1 (4)	3700
Mesothelioma	1 (4)	14,726
Mastocytes	1 (4)	3108

**Table 3:** Univariable predictors of SPM.

	Hazard Ratio (95% CI)	*p* Value
Age at the time of DTC diagnosis	1.049 (1.021–1.078)	0.001
Age ≥ 55 years	3.990 (1.792–8.880)	0.001
Female gender	0.658 (0.275–1.575)	0.35
Papillary type	1.183 (0.537–2.607)	0.68
>T2	1.954 (0.733–5.206)	0.18
N1	0.036 (0.001–2.733)	0.13
Thyroglobulin level at first RAI	1.000 (0.997–1.002)	0.73
^131^I cumulative activity dose	1.001 (0.998–1.003)	0.58
<1850 MBq	0.352 (0.048–2.600)	0.31
1850–3700 MBq	0.617 (0.280–1.359)	0.23
>3700 MBq	0.895 (0.863–4.159)	0.11
Follow-up duration	1.007 (1.002–1.011)	0.001

## Data Availability

The data presented in this study are available on request from the corresponding author. The data are not publicly available due to privacy restrictions.
